# Pathogenic Variants in *ABHD16A* Cause a Novel Psychomotor Developmental Disorder With Spastic Paraplegia

**DOI:** 10.3389/fneur.2021.720201

**Published:** 2021-08-20

**Authors:** Ashraf Yahia, Liena E. O. Elsayed, Remi Valter, Ahlam A. A. Hamed, Inaam N. Mohammed, Maha A. Elseed, Mustafa A. Salih, Typhaine Esteves, Nicolas Auger, Rayan Abubaker, Mahmoud Koko, Fatima Abozar, Hiba Malik, Rawaa Adil, Sara Emad, Mhammed Alhassan Musallam, Razaz Idris, Isra Z. M. Eltazi, Arwa Babai, Elhami A. A. Ahmed, Amal S. I. Abd Allah, Mathilde Mairey, Ahmed K. M. A. Ahmed, Mustafa I. Elbashir, Alexis Brice, Muntaser E. Ibrahim, Ammar E. Ahmed, Foudil Lamari, Giovanni Stevanin

**Affiliations:** ^1^Faculty of Medicine, University of Khartoum, Khartoum, Sudan; ^2^Department of Biochemistry, Faculty of Medicine, National University, Khartoum, Sudan; ^3^Sorbonne Université, Institut du Cerveau–Paris Brain Institute, INSERM, CNRS, Hôpital Pitié-Salpêtrière, Paris, France; ^4^Ecole Pratique des Hautes Etudes, EPHE, PSL Research University, Paris, France; ^5^College of Medicine, Princess Nourah Bint Abdulrahman University, Riyadh, Saudi Arabia; ^6^Division of Pediatric Neurology, Department of Pediatrics, College of Medicine, King Saud University, Riyadh, Saudi Arabia; ^7^Department of Molecular Biology, Institute of Endemic Diseases, University of Khartoum, Khartoum, Sudan; ^8^Department of Neurology and Epileptology, Hertie Institute for Clinical Brain Research, Tubingen, Germany; ^9^Letterkenny University Hospital, Letterkenny, Ireland; ^10^UNESCO Chair on Bioethics, University of Khartoum, Khartoum, Sudan; ^11^Department of Molecular Neuroscience, Graduate School of Medicine, Osaka University, Suita, Japan; ^12^Immunology Frontier Research Center, Osaka University, Suita, Japan; ^13^APHP, Pitié-Salpêtrière Hospital, Metabolic Biochemistry unit, Department of Biochemistry of Neurometabolic Diseases, Paris, France

**Keywords:** *ABHD16A*, hereditary spastic paraplegia, next-generation sequencing, targeted-metabolomics, lipid metabolism, phosphatidylserine

## Abstract

**Introduction:** Hereditary spastic paraplegia is a clinically and genetically heterogeneous neurological entity that includes more than 80 disorders which share lower limb spasticity as a common feature. Abnormalities in multiple cellular processes are implicated in their pathogenesis, including lipid metabolism; but still 40% of the patients are undiagnosed. Our goal was to identify the disease-causing variants in Sudanese families excluded for known genetic causes and describe a novel clinico-genetic entity.

**Methods:** We studied four patients from two unrelated consanguineous Sudanese families who manifested a neurological phenotype characterized by spasticity, psychomotor developmental delay and/or regression, and intellectual impairment. We applied next-generation sequencing, bioinformatics analysis, and Sanger sequencing to identify the genetic culprit. We then explored the consequences of the identified variants in patients-derived fibroblasts using targeted-lipidomics strategies.

**Results and Discussion:** Two homozygous variants in *ABHD16A* segregated with the disease in the two studied families. *ABHD16A* encodes the main brain phosphatidylserine hydrolase. *In vitro*, we confirmed that *ABHD16A* loss of function reduces the levels of certain long-chain lysophosphatidylserine species while increases the levels of multiple phosphatidylserine species in patient's fibroblasts.

**Conclusion:***ABHD16A* loss of function is implicated in the pathogenesis of a novel form of complex hereditary spastic paraplegia.

## Introduction

Hereditary spastic paraplegia (HSP) is an entity that includes a heterogeneous group of disorders characterized clinically by spastic lower limbs, either alone (pure HSP) or in combination with other neurological and/or extra-neurological features (complex HSP) (1). Intellectual impairment, cerebellar ataxia, neuropathy, and skeletal deformities are common presentations complicating HSP (2). The advent of next-generation sequencing (NGS) has revolutionized HSP diagnosis and emphasized its extreme genetic heterogeneity broadening the list of the currently identified HSP-causing genes to include more than 80 genes (3). The HSP global prevalence has been estimated as 1.8/10^5^(4), a value probably underestimated because of the lack of accessibility to new diagnostic technologies (3, 4).

Multiple pathological mechanisms contribute to the development of HSP. These mechanisms affect cellular processes involved in the corticospinal tract's neurodevelopment and maintenance, including dysfunctions of the endoplasmic reticulum (ER), mitochondria, cytoskeleton, cell signaling pathways, neuronal development, and cellular metabolism (5, 6). Accumulating evidence emphasizes the importance of maintaining lipid homeostasis for the health and function of neurons (7), particularly in HSP forms.

Lipids constitute approximately one- to two-thirds of the gray matter, white matter, and myelin in the human brain, where they have structural and signaling functions (8–12). Glycerophospholipids (GPL) constitute a major class of lipids, composed of a glycerol backbone linked at its *sn*-1 and *sn*-2 carbons to two fatty acid chains and at its *sn*-3 carbon to a polar head group (13, 14). GPL lacking one long-chain fatty acid attached to its glycerol backbone are called lysophospholipids (13).

The brain is highly enriched in GPL compared to other organs (15). The most abundant classes are phosphatidylethanolamine (55%), phosphatidylcholine (31%), and phosphatidylserine (PS, 7.7%) (15). Disturbances in the GPL biosynthesis and remodeling pathways are increasingly implicated in the pathogenesis of multiple HSP subtypes (7).

Herein we report a form of complicated HSP caused by homozygous variants in the *ABHD16A* gene involved in PS metabolism.

## Materials and Methods

We used genetic and functional tools to reach the cause of the disease in the two consanguineous Sudanese families F37 and F69. Supplementary Figure 1 summarizes the approaches we followed in our quest; the detailed methodology is provided below.

### Clinical Phenotyping and Sampling

This study was approved by local Ethics committees, and signed consent was obtained for all patients. The patients were evaluated at the Pediatric Neurology Clinic, Soba University Hospital (Khartoum, Sudan). We collected two ml of saliva from the patients and healthy related controls using OG-500 or OG-575 Oragene DNA kits (DNA Genotek Inc., Ottawa, ON, Canada). DNA was extracted according to the PrepIT L2P protocol provided by the manufacturer.

Skin punch biopsies were obtained from patients F37-314, F37-315, and F69-508 and unrelated healthy Sudanese controls using 4-mm-punches. Immediately after the biopsy, samples were preserved in 20 ml sterile clear glass vials filled with a culture medium composed of 50% Gibco™ DMEM (Dulbecco's Modified Eagle Medium), 10% Gibco™ L-Glutamine (l x solution), 10% Penicillin/Streptomycin (P 4333 Sigma-Aldrich, l x solution), 10% Gibco^TM^ HEPES [4-(2-hydroxyethy1)-1-piperazineethanesulfonic acid], and 20% Gibco™ fetal bovine serum. Punches were sent at room temperature for fibroblasts culturing at the platform of biological resources of the Paris Brain Institute–ICM, Paris, France.

#### Next-Generation Sequencing

DNA extracted from patients F37-314, F37-315, F69-508, and F69-509 was sent for NGS at the genotyping and sequencing core facility of the Paris Brain Institute -ICM, Paris, France. First, we screened the DNA for pathogenic variants in known HSP genes using a targeted next-generation sequencing panel surveillance of 65 genes as detailed in a previous report (16). Following negative panel surveillance, exome sequencing (ES) was used for the analysis of the DNA of three patients (F37-314, F69-508, and F69-509). ES data were generated and processed as described previously (17).

#### Sanger Sequencing

Sanger sequencing was performed on an ABI3730 sequencer (Applied Biosystems) using the Bigdye chemistry at GATC Biotech—Eurofins Genomics (Cologne, Germany). Segregation analysis of variants was performed in all available samples from the two families.

#### Cell Culture

Fibroblasts were prepared at the density of 2 x 10^7^ cells/100 mm culture plate, washed three times with 1 × Phosphate Buffer Saline (PBS), and then cultured for 4 h in 5 ml of High Glucose Dulbecco's Modified Eagle Medium (DMEM) 4.5 g.L-1 (Gibco) (for lipidomic studies, the medium contained neither phenol red nor serum to avoid interference). We centrifuged the cells-containing medium (4,500 g, 20°C, 5 min) and stored the supernatant at −80°C before lipids extraction. We detached the cells from the medium using trypsin, then centrifuged the mix (800 rpm, 20°C, 5 min), washed it with PBS three times, and froze (−80°C) the resulting pellet.

#### Protein Extraction and Quantification

The cells were detached with 0,05% trypsin (Gibco) in PBS, centrifuged (1,000 rpm, 20 °C, 5 min), and washed with 1 × PBS. The resulting cell pellet was lysed in lysis buffer (100 mM NaCl, 10 mM Tris Hcl pH 7.4, 1% Triton X-100, and 1 × protease inhibitors–HaltTM Protease Inhibitor Cocktail 100 ×) (Thermo Scientific). After 30 min of incubation on ice, the lysates were centrifuged (13,000 rpm, 4 °C, 30 min), and the supernatants were collected.

The protein assay was performed with the “BCA Protein Assay Kit” (Pierce^TM^), allowing the quantification of copper reduction caused by proteins in alkaline solution, with a colorimetric detection (absorbance at 562 nm) of the bicinchoninic acid reduced Cu^+1^ cation.

We compared the absorbance obtained to the absorbance values of a Bovine Serum Albumin (BSA) standard with a 0–20 μg.mL-1 range to deduce our samples' protein concentration.

#### Detection of Proteins by Western Blot

Protein samples were mixed with Laemmli loading buffer (2% SDS, 10% Glycerol, 60 mM Tris-HCl pH 6.8, 0.01% Bromophenol blue, and 50 mM DTT), then denatured for 5 min at 95°C. Ten to fifteen micrograms of proteins were deposited on a precast acrylamide gel “NuPAGE™ 4 to 12%, Bis-Tris, 1.0 mm, Mini Protein Gel, 10-well” or “Novex 4–20% Tris-glycine mini protein gel” (ThermoFisher Scientific) with the molecular weight scale “PageRulerTM Plus Prestained Protein Ladder” (ThermoFisher Scientific). Migration was performed at 120–150 V constant in MOPS migration buffer (50 mM MOPS, 50 mM Tris-base, 1 mM EDTA and 0.1% SDS). A “semi-dry” transfer was carried out on a nitrocellulose membrane (Amersham™ Protran® 0.45 NC) for 60–90 min at 25 V constant and 0.22 A per membrane in transfer buffer (25 mM Tris-base, 292 mM Glycine, 20% ethanol, 0.1% SDS). The membrane was then incubated in Ponceau red to check transfer efficiency and washed by PBS.

The membrane was incubated in a saturation solution (1 × PBS, 5% milk, and 5% BSA) for 1 h with shaking at room temperature, then incubated overnight at 4°C in the saturation solution with the primary anti-ABHD16A (Abcam® EPR15463) or anti-alpha tubulin antibody (Abcam® ab7291). The membrane was then washed three times in 1 × PBS 0.1% Tween for 5 min, incubated for 1 h in saturation solution with anti-rabbit (IRDye® 800 Donkey anti-Rabbit IgG) or anti-mouse secondary antibodies (IRDye® 680 Goat anti-Mouse IgG) then washed three times with 1 × PBS. The detection was carried out by reading fluorescence with the Odyssey® Clx or with SuperSignal™ West Femto Maximum Sensitivity Substrate (ThermoFisher Scientific) at ChemiDoc™ system. The analysis of the images obtained was carried out with ImageJ software.

#### Extraction of Phospholipids

We extracted lipids from cells using the Bligh & Dyer method (18). The cell pellets obtained from the preparation steps were suspended in 1 ml of PBS, and 100 μl of each suspension was stored for protein quantification. The cell samples were placed in glass tubes and supplemented with 1 μg of LPS 17:1 as internal standard (Avanti® Polar Lipids, USA).

Lipids were extracted twice by adding 3 ml of chloroform/methanol (2:1, v/v). The lipid extract was evaporated to dryness under a stream of nitrogen, the residues were dissolved in 100 μl of chloroform/methanol (2:1, v/v), and 10 μl were injected into the LC-MSMS system. For PS quantification, a five-point calibration curve was made by a successive dilution of a solution mixture of Phosphatidyl-L-serine from bovine brain (Sigma Aldrich).

#### Ultra Performance Liquid Chromatography/Mass Spectrometry Procedure and Analysis

Separation of PS and LPS were carried on ultra-performance liquid chromatography (Acquity-Waters) equipped with a Waters-Acquity UPLC BEH C8 (1.7 μm, 2.1 ×100 mm) column at 60 °C. Mobile phases were: phase A: water/ methanol (95:5) (v/v) supplemented with 0.1% formic acid 0.028% ammonia and 5 μM phosphoric acid; mobile phase B: iPrOH/ MeOH/H_2_O (60:35:5, v/v/v) supplemented with 0.1% formic acid and 0.028% ammonia. PS and LPS detection and quantification were performed on a TQD mass spectrometer (Waters) equipped with electrospray ionization. The spectra were recorded in MRM, negative ion mode (Supplementary Table 1). The capillary voltage was set at 3 kV, cone voltage at −40 V, and source block temperature 130 °C.

## Results

### Clinical Presentation

#### Family F37

**Figure 1:**
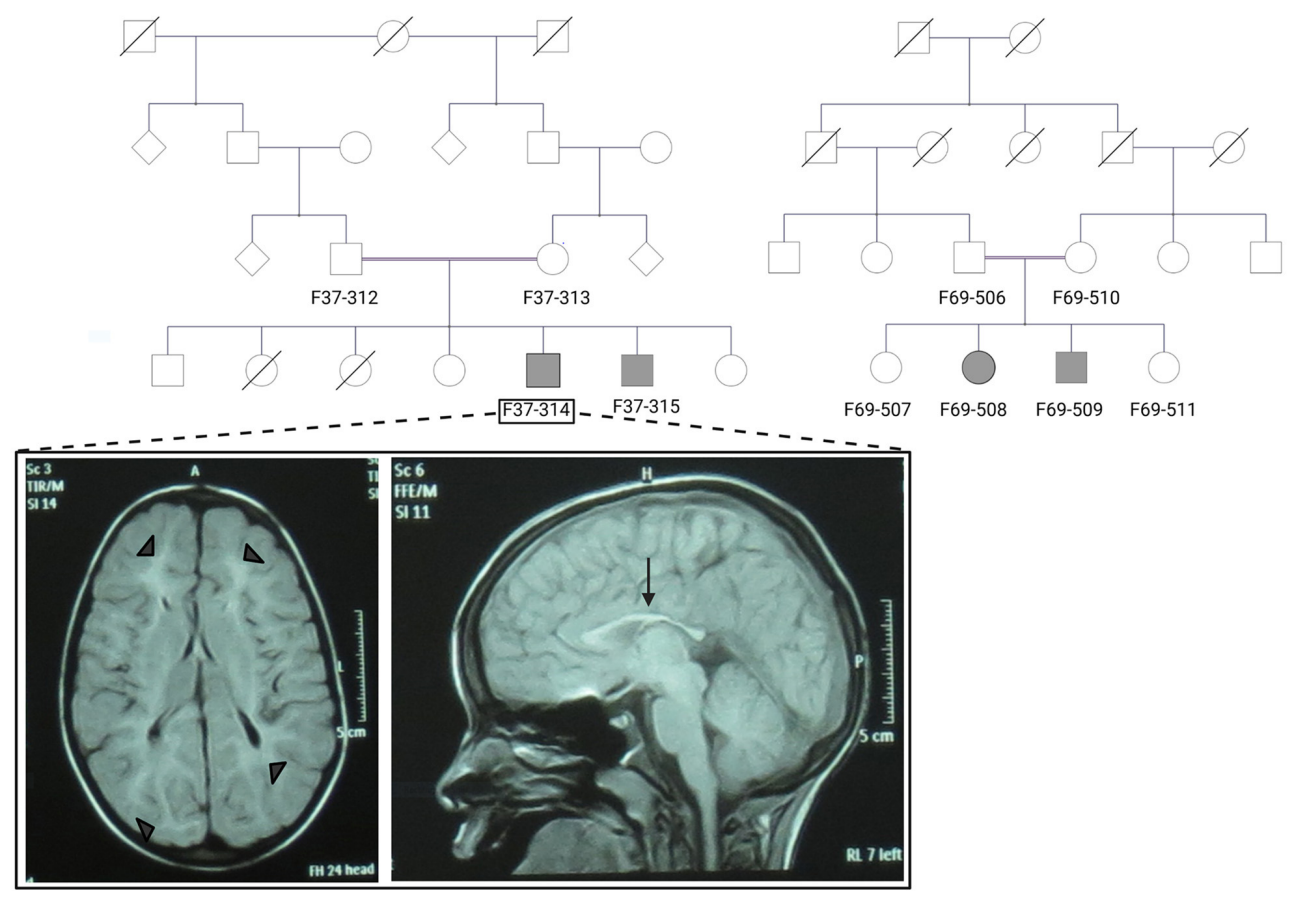
The pedigrees of families F37 and F69 and patient F37-314 brain MRI. The family pedigrees of family F37 (left) and F69 (right) show consanguinity in the two families. Axial T2 TIRM dark fluid brain MRI of patient F37-314 shows periventricular and subcortical white matter hyperintensities (left image: arrow heads), whereas sagittal T1 image shows thin corpus callosum (right image: arrow).

Patients F37-314 (10 years) and F37-315 (7 years) were male siblings born to first-degree consanguineous Sudanese parents after uneventful pregnancies and deliveries (Figure 1). The two boys manifested a neurodevelopmental disorder with a comparable disease progression. Their disease was characterized by the absence of comprehensible speech, stiffening of their lower limbs observed after their first birthday, and intellectual impairment (Table 1).

**Table 1:** Summary of the clinical presentation of the patients from the families F37 and F69.

**Patient identifier**	**F37-314**	**F37-315**	**F69-508**	**F69-509**
Gender	Male	Male	Female	Male
Age at examination (years)	10	7	8	7
Age at onset	Early childhood (<1 year)	Early childhood (<1 year)	5 months	1 week
Initial presentation	Developmental delay and LL stiffness	Developmental delay and LL stiffness	Delayed development after a febrile illness	Delayed development after a febrile illness
Intellectual disability	Present	Present	Present	Present
Walking	Wheel chair-dependent	Wheel chair-dependent	With support	With support
Speech	Absent	Absent	Absent	Absent
Convulsions	Absent	Absent	Twice (febrile)	Absent
Others symptoms	Psychiatric features	Psychiatric features	–	–
Skeletal deformities	–	Bilateral pes equinovarus	Neurological examination	Scoliosis and bilateral pes equinovalgus
Wasting	Mild in the proximal LL	Moderate in the LL	Scoliosis, bilateral pes cavus, and pes equinovalgus	Absent
Spasticity	LL	LL	UL and LL	UL and LL
Power	Normal proximally; Not assessed distally	Normal proximally; Grade 3 distally	Grade 3 in the lower limbs	Grade 3 in the lower limbs
Hyperreflexia	Bilateral Patellar and adductor	Bilateral Biceps, finger flexors, patellar, adductor, and ankle	Bilateral patellar, adductor, ankle, and biceps	Bilateral biceps, triceps, finger flexors, patellar, adductor, and ankle
Babinski's sign	Not examined	Negative	Positive	Positive
Cerebellar signs	Absent	Absent	Absent	Absent
Extrapyramidal features	Choreoathetosis	Choreoathetosis	Absent	Absent
Brain MRI	TCC, subcortical, and periventricular WMH	TCC, subcortical, and periventricular WMH	Not done	Not done

*UL, upper limbs; LL, lower limbs; MRI, magnetic resonance imaging; TCC, thin corpus callosum; WMH, white matter signal hyperintensities*.

They sustained head control at 4 months, sat without support at 9 months, and had their first social smiles at 4 months. Both never spoke, except for some incomprehensible sounds. The older sibling (F37-314) started to walk at 1 year, while the younger walked at two, though their gait was abnormal due to spasticity of the lower limbs. At 3–4 years, their walking and speaking skills regressed; patient F37-314 reverted to crawling, while patient F37-315 only walked with support. They acquired sphincter control at 2 and 3 years of age (younger and older sib, respectively), and maintained sphincter control as their condition progressed. At the ages of seven and six, respectively, they became non-ambulant and wheelchair-dependent.

The upper limb examination of the two siblings was normal. Lower limb examination was significant for bilateral spasticity and hyperreflexia. We could not examine the older patient's ankle and distal lower limb because his legs were in a cast after corrective surgery for ankle deformity at the time of examination. The younger patient (F37-315) had talipes equinovarus, more pronounced on the left leg, and bilateral up-going plantar response. The younger patient had mild to moderate wasting of the lower limbs with preferential decrease in the strength distally and sparing of proximal lower limb motor function. Coordination was normal, we did not perform heel-on-shin test in the older sibling, and there was no oculomotor apraxia. Fundal examinations were normal. Due to a lack of cooperation, we could not examine their sensory system. We noted extrapyramidal signs in form of choreoathetosis in the two siblings. It was evident that both siblings had cognitive impairment and learning difficulties but no formal neuropsychology assessment was performed in this study. They were irritable, distractible, had bouts of crying, and had emotional lability. We also noted a stereotypic behavior in the form of hand clapping and head banging.

Brain Magnetic resonance imaging (MRI) showed thin corpus callosum and subcortical and periventricular white matter high signals in both cases (Figure 1).

#### Family F69

The patients F69-508 and F69-509 were two Sudanese siblings aged 8 and 7 years, respectively (Table 1). They suffered from a neurodevelopmental disorder that was noted to start following a febrile illness. Both patients were outcomes of unremarkable pregnancies ending by normal vaginal deliveries. The parents were second-degree relatives; however, there was no family history of a similar condition (Figure 1).

Patient F69-508 was a healthy baby with normal development until she had attacks of febrile convulsions between the ages of 5 months and 1 year. After these episodes, all categories of developmental milestones regressed. She walked with support at the age of 4 years, and until the age of presentation, 8 years old, she could neither walk independently nor speak, apart from a few incomprehensive sounds. Her intellectual development was markedly delayed for her age, and she never controlled her sphincters. On examination, her head circumference was 51.5 cm, which is within the normal range for her age. Examination of her limbs was significant for spasticity, severe in the lower limbs and mild in the upper limbs, hyperreflexia, and bilateral up-going plantar response. Her patellar, adductor, ankle, and biceps reflexes were increased. She had normal finger flexor reflex and an absent jaw jerk. Hoffmann's sign was negative. She presented with skeletal abnormalities such as mild scoliosis, severe pes cavus, and pes equinovarus. Ocular motor and cerebellar examination were normal. We did not perform a fundus examination. Although a formal sensory examination was not possible, we did not identify any major sensory impairment.

The patient F69-509's disease started 1 week after birth with a febrile illness not complicated with convulsions. Following the febrile illness, the patient became hypotonic. Akin to his sister, patient F69-509 progressed to have global developmental impairment. He started to walk with support at 4 years and never walked unsupported. Although he could say more words compared to his sister, he never formulated a sentence. He had cognitive impairment and did not achieve sphincter control.

On clinical examination, he had severe spasticity in upper and lower limbs, hyperreflexia, and bilateral up-going plantar responses. The power was reduced in his lower limbs though he had a normal overall muscle bulk. The patient F69-509 had scoliosis and pes equinovalgus, and, similar to his sister, he had a normal head circumference for his age (51 cm). Ocular motor and cerebellar examinations were normal. Brain MRI was not available for the two affected children.

### Genetic Results

In the absence of genetic variants in targeted sequencing of 65 HSP genes (16), we performed ES on the patients from families F37 (F37-314) and F69 (F69-508 and F69-509) with a mean coverage of 30 and 100 × , respectively. Filtering of variants on ES data identified homozygous variants in *ABHD16A* as the only convincing variants.

In family F37, the nonsense variant NM_021160.2(*ABHD16A*):c.340C>T (p.Arg114^*^) was confirmed as homozygous in patients F37-314 and F37-315 using Sanger sequencing. We validated its segregation with the disease in the family F37 under an autosomal recessive model of inheritance. This variant was absent in the gnomAD v2.1.1 database and the Greater Middle East Variome project.

In family F69, the missense variant NM_021160.2(*ABHD16A*):c.1370G>A (p.Arg457Gln) (rs774259910) was confirmed homozygous in the patients F69-508 and F69-509 and segregated with the disease under an autosomal recessive model of inheritance. While this variant was absent in the Greater Middle East Variome project, it was found extremely rare in the gnomAD v2.1.1 population as it was reported only once in a heterozygous state in an African/African American male (its global gnomAD frequency was 0.000004). NM_021160.2(*ABHD16A*):c.1370G>A (p.Arg457Gln) was predicted to alter a highly conserved Arginine at position 457 (CADD score 36). Furthermore, NM_021160.2(*ABHD16A*):c.1370G was the last base before the splice site in *ABHD16A*'s exon 16, and its disruption was predicted to alter *ABHD16A* mRNA splicing. We employed two splice-effect prediction algorithms, MaxEntScan and NNSPLICE to scan for novel splice sites. MaxEntScan uses the Maximum Entropy principle to identify splicing motifs (19). On the other hand, NNSPLICE employs a neural network model to recognize splice sites (20). The MaxENTScan score (0–12) was 8.51 for the wild-type variant and 2.69 for the mutant, while the NNSPLICE score (0–1) moved from 0.93 for the wild-type variant to 0 for the mutant.

We found no other HSP family carrying loss of function pathogenic variants in our in-house exome database of 450 exomes of ataxia and HSP cases and in the GENESIS platform (https://www.tgp-foundation.org/genesis-log-in) gathering 786 exomes of pure and complex spastic paraplegia.

### Functional Studies

#### ABHD16A Protein Was Present in Controls' but Not Patients' Fibroblasts

We checked for the presence of the ABHD16A protein in the fibroblasts of three affected patients and two unrelated controls. While the positive control protein, alpha-tubulin, was present in all the examined fibroblasts, ABHD16A was absent in the fibroblasts derived from the patients F69-508, F37-314, and F37-315 and was present in the fibroblasts obtained from the two unrelated healthy controls (Supplementary Figure 2). This corroborated the predictions that both NM_021160.2(*ABHD16A*):c.340C>T (p.Arg114^*^) and NM_021160.2(*ABHD16A*):c.1370G>A (p.Arg457Gln) were null variants.

#### PS Levels Were Higher While LPS Levels Were Lower in Patients' Compared to Controls' Fibroblasts

**Figure 2:**
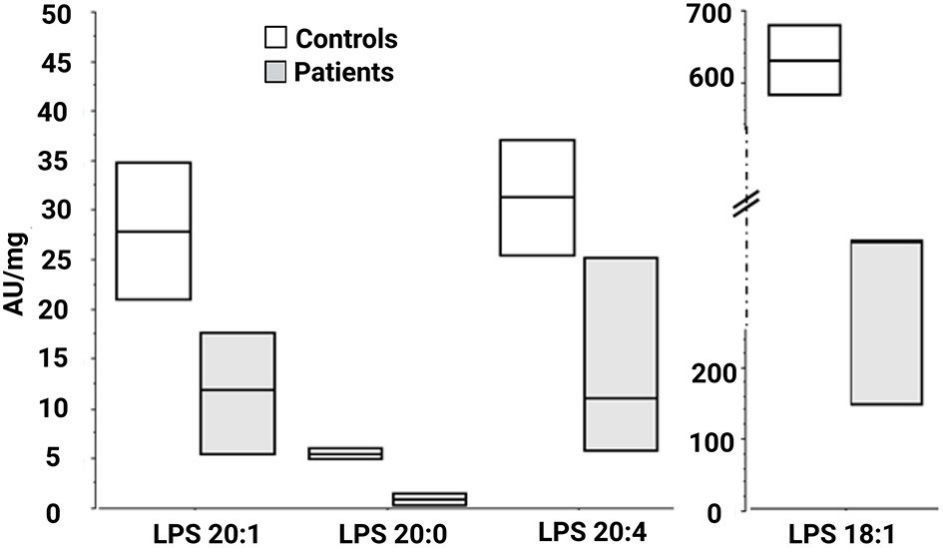
Lysophosphatidylserine molecular species in fibroblasts derived from patients F37-314, F37-315, and F69-508. LPS 18:1, 20:0, 20:1, and 20:4 are lower in the patients than in the healthy controls. AU, arbitrary units; LPS, lysophosphatidylserine.

We then looked for the consequences of the absence of ABHD16A in fibroblasts of patients by measuring PS/LPS using UPLC\MS. The levels of the main detected PS species, expressed as arbitrary units/mg proteins, were systematically higher in fibroblasts from the patients F37-314, F37-315, and F69-508 compared to cells from unrelated healthy controls (Table 2). However, the difference between the patients and the controls' cells was statistically significant for only 34:1, 36:2, 36:1, and 42:1 species (Table 2, *p* = 0.014), likely due to the low sample size. In contrast, the levels of long-chain LPS (18:1, 20:4, 20:0, and 20:1) were reduced in the patient's fibroblasts compared to control cells (Figure 2, *p* < 0.0001). These results, suggest that ABHD16A pathogenic variants are loss of function mutations, leading to accumulation of several PS substrate species and reduction of several LPS product species.

**Table 2:** Phosphatidylserine molecular species in fibroblasts derived from the patients F69-508, F37-314, and F37-315 and two unrelated healthy controls.

**Individual PS species**	**F37-314**	**F37-315**	**F69-508**	CTRL^*^ (mean ± SD)
PS 34:1	46.2	34.5	49.4	24.1 ± 2.2
PS 36:2	4.9	4.3	4.5	3.2 ± 0.2
PS 36:1	9.6	6.2	8.7	4.3 ± 0.4
PS 38:1	5.8	2.2	3.0	1.2 ± 0.3
PS 40:8	7.8	1.5	3.0	0.9 ± 0.1
PS 40:7	3.6	0.8	2.2	0.5 ± 0.2
PS 40:6	2.9	2.5	1.7	1.5 ± 0.2
PS 40:5	22.4	10.6	7.5	4.9 ± 0.5
PS 40:4	17.6	6.0	8.0	2.9 ± 0.2
PS 40:3	4.0	1.1	1.6	0.6 ± 0.1
PS 40:1	9.3	2.8	4.6	1.3 ± 0.2
PS 42:2	9.1	2.4	4.5	1.6 ± 0.2
PS 42:1	28.4	19.1	14.9	7.4 ± 1.1
PS 44:1	17.8	10.1	4.3	4.6 ± 2.3

*Data are expressed as arbitrary units/mg proteins*.

* *Control fibroblasts were obtained from two unrelated healthy controls. PS, Phosphatidylserine*.

## Discussion

We identified two homozygous null variants in the *ABHD16A* gene segregating in two Sudanese families, causing a novel neurodevelopmental disorder. The clinical presentation in the patients from the two families was severe and included developmental delay, intellectual impairment, spasticity, and skeletal deformities, reminiscent of complex forms of hereditary spastic paraplegia.

*ABHD16A* is a 21-exon gene which encodes for the α/β hydrolase domain-containing 16A (ABHD16A) enzyme, which is the main brain phosphatidylserine (PS) hydrolase (21). It has two protein-coding transcripts, NM_021160 (the most ubiquitous) and NM_001177515, which code for two functionally related isoforms of the ABHD16A enzyme, with 558 and 525 amino acids length, respectively (22, 23). NM_021160 is highly expressed in the brain, muscles, testes, and heart, while the highest expression of NM_001177515 is in the testes, with still a minimal expression in the brain (22, 23). The two variants identified in this study are predicted to have the same effect on the two *ABHD16A* transcripts, leading to their complete loss of function; we focused our study on the canonical and most ubiquitous transcript, NM_021160.

**Figure 3:**
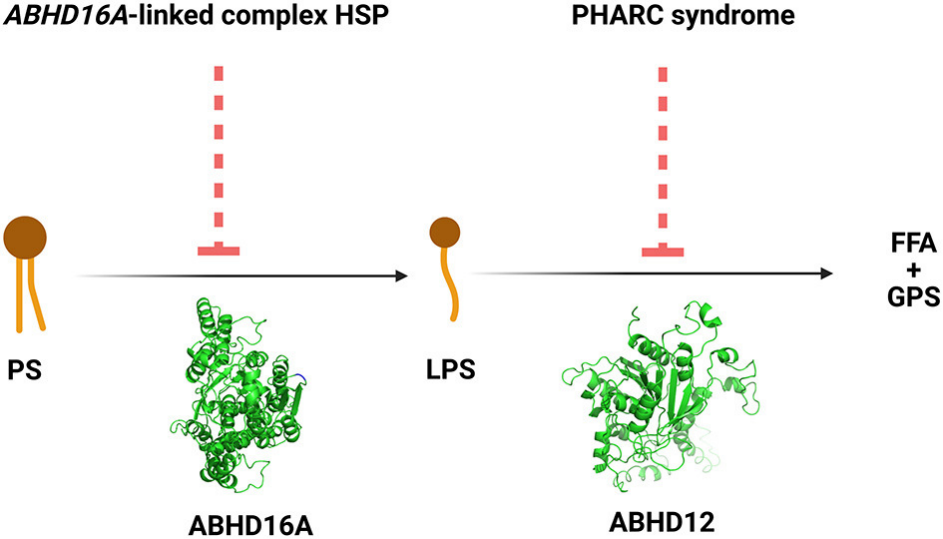
The reactions and the disorders across the ABHD16A-ABHD12 pathway. HSP, hereditary spastic paraplegia; PHARC syndrome, syndrome of polyneuropathy, hearing loss, ataxia, retinitis pigmentosa, and cataract; PS, phosphatidylserine; LPS, lysophosphatidylserine; FFA, free fatty acid; GPS, glycerophosphoserine.

The ABHD16A enzyme is a member of the α/β hydrolase domain-containing protein family that participates in lipid metabolism and intracellular signaling (23). Pathogenic variants in genes coding for enzymes belonging to α/β hydrolases family have already been linked to inherited metabolic diseases. This is the case for *ABHD5* and *ABHD12* causing Chanarin-Dorfmann (OMIM # 275630) and polyneuropathy, hearing loss, ataxia, retinitis pigmentosa, and cataract (PHARC; OMIM # 612674) syndromes, respectively (24, 25). *ABHD16A* is highly conserved in mammals (22, 23). *ABHD16A* has a heterogeneous expression in the murine brain and is enriched in the cerebellum, particularly in the granular layer and the olfactory bulb (26). It is an integral ER protein with a cytosolically oriented active site (26). In the ER, it colocalizes with the principle brain LPS hydrolase ABHD12, responsible for the PHARC syndrome (OMIM # 612674) (25–27). ABHD12, unlike ABHD16A, is ubiquitously expressed in the brain (26). These two enzymes, ABHD16A and ABHD12, are the main enzymes responsible for the brain LPS metabolism, as ABHD16A generates LPS while ABHD12 catabolizes them into fatty acid and phosphoserine (Figure 3).

The PS substrates for the ABHD16A-ABHD12 pathway are continuously replenished from other phospholipid precursors, particularly at the mitochondrial-associated membranes (28). PS is the major acidic phospholipid class in the brain, accounting for >7% of the total brain phospholipids (15). It participates in multiple neuronal processes, including synaptic formation, synaptic functions, and neuronal signaling (28–30). Pathogenic variants in genes coding certain enzymes involved in PS metabolism have been associated with human genetic disorders. For example, gain-of-function pathogenic variants in *PTDSS1* coding phosphatidylserine synthase causes Lens-Majewski syndrome (OMIM # 151050) characterized by skeletal deformities and intellectual disability; similarly, pathogenic variants in *PISD* coding phosphatidylserine decarboxylase were associated with Liberfarb syndrome (OMIM # 618889), also characterized by skeletal deformities (31, 32).

LPS, the deacylated derivative of PS, participates in cellular signaling and has at least three receptors in humans, namely G-protein-coupled receptor (GPR) 34, GPR 174, and the purinergic receptor P2Y-G-Protein-Coupled 10 (33–37). In addition, LPS potentiates the effects of neuronal growth factor (NGF) in causing PC12 cells to differentiate into primary neurons (38). The combined actions of NGF and LPS are important for acquiring and maintaining the unipolar and bipolar morphology of the primary neurons (38). However, the mechanism by which LPS potentiates the effects of NGF is not determined yet (38).

PS and LPS levels are altered to variable extents across the brains of *ABHD16A* and *ABHD12* knockout mice (26). Loss of ABHD16A activity in mice causes a decrease in brain LPS, particularly in the cerebellum and olfactory bulb (26). In this model, the reduced LPS species are mostly those with long-chain fatty acids (C18 to C22), and this is in line with what we observed in our patients' fibroblasts. Regarding the peripheral, more accessible tissues, a reduction in ABHD12 activity was documented in lymphoblastoid cells from a human patient with PHARC syndrome compared to healthy controls (39).

To explore the signatures of ABHD16A deficiency in human fibroblasts and its contribution to the novel phenotype identified in the patients from the families F37 and F69, we first, validated the lack of expression of ABHD16A in patients' fibroblasts vs. controls. We then measured PS and LPS in the fibroblasts derived from three patients with *ABHD16A* variants and two healthy controls using UPLC\MS. We found the levels of most PS species higher in the *ABHD16A* patients' cells, particularly those with long-chain fatty acids compared to healthy controls' cells. These results are in line with what has been reported in the *ABHD16A* knockout mice model and confirm the loss of function of the ABHD16A protein and its specificity against long chains PS.

In the ABHD16A^−/−^ mouse model that was established by Kamat et al. they could identify sub-Mendelian production of the mice which was explained by presumed prenatal lethality (21).

The pathogenic mechanisms of ABHD16A deficiency could be due to the loss of LPS potentiation of NGF-mediated neuronal development or another yet unidentified LPS-mediated signaling. ABHD16A deficiency is associated with low brain LPS levels; thus, in contrast to PHARC syndrome, the pathogenesis of the *ABHD16A*-associated HSP is unlikely due to LPS-mediated activation of microglia and neuroinflammation (26). The pathogenic mechanism can also be related to PS metabolism. Indeed, gain-of-function pathogenic variants in *PTDSS1* coding PS synthase cause sclerosing bone dysplasia and intellectual disability (Lens-Majewski syndrome; OMIM # 151050), and pathogenic variants in *PISD1* coding PS decarboxylase cause Spondyloepimetaphyseal dysplasias (31, 32). Interestingly, the patients from both F37 and F69 families exhibited skeletal deformities and had intellectual disabilities. Further studies are needed to elucidate the pathogenesis of the *ABHD16A*-associated HSP and delineate its associated phenotype. Fibroblasts - and the study of their PS/LPS contents by MS–can provide a means for establishing the diagnosis of future patients with the novel *ABHD16A*-linked complex HSP, particularly those with missense variants.

Important therapeutic options may emerge based on the association of defects in ABHD16A with HSP and neurodevelopmental phenotype and low levels of brain LPS identified in our current study. The pathogenic mechanisms of *ABHD16A*-associated HSP are yet to be fully revealed; however, given the interplay of the two lipases along the ABHD16A-ABHD12 axis, normalization of low LPS level can be implemented in treating *ABHD16A*-associated HSP using selective ABHD12 inhibitors. Although a number of ABHD12 inhibitors are known, they are mostly non-selective. However, a study identified DO264 as an *in-vivo* selective inhibitor of ABHD12, elevating LPS in a dose dependent manner in human macrophages and mouse brain (21). Another study constructed a model for some triterpenes-based ABHD12 reversible inhibitors (40). The model was based on results of provisional screening that identified selective inhibition of ABHD12 by ursolic acid with negligible non-selectivity (40). The provisional structure-activity relationships that the study established can pave the way for discovery of more selective ABHD12 inhibitors that may be target for treatment of ABHD16A-associated HSP.

In conclusion, we identified a novel form of complicated HSP caused by *ABHD16A* loss of function pathogenic variants and the subsequent altered PS metabolism. Our study emphasizes the role of lipid metabolism and the ABHD16A-ABHD12 axis in HSP; though, the data on the pathogenesis of *ABHD16A*-associated HSP is still limited and requires functional experiments to link PS/LPS levels to neurodegeneration. We propose that lipidomics could be used to validate the functional effect of *ABHD16A* missense variants in the future, often a bottleneck in genetic studies.

## Data Availability Statement

The datasets presented in this study can be found in online repositories. The names of the repository/repositories and accession number(s) can be found at: https://www.ncbi.nlm.nih.gov/clinvar/, SCV001653694; SCV001653696.

## Ethics Statement

The studies involving human participants were reviewed and approved by The Ethical Committee of Medical Campus, University of Khartoum, Sudan and The Ethical Committee of National University, Sudan (approval number NU-RECG200). Written informed consent to participate in this study was provided by the participants' legal guardian/next of kin. Written informed consent was obtained from the individual(s), and minor(s)' legal guardian/next of kin, for the publication of any potentially identifiable images or data included in this article.

## Author Contributions

AY, LE, MIE, FL, and GS: conceptualization. AY, LE, RAb, MK, RI, IE, ABa, EA, AAb, MMa, AAh, FA, HM, RAd, SE, and MMu: data curation. AY, LE, and RV: formal analysis. ABr, MIE, AEA, and GS: funding acquisition. AY, LE, AH, IM, MAE, MS, TE, NA, RAb, MK, RI, IE, ABa, EA, AAb, MMa, AAh, FA, HM, RAd, SE, MMu, and FL: investigation. AY, LE, RV, TE, NA, FL, and GS: methodology. ABr, MIE, MI, AEA, and GS: project administration. ABr, AH, IM, MAE, MS, FL, and GS: resources. MIE, MI, AEA, and GS: supervision. AY, FL, and GS: validation. AY, LE, RV, TE, NA, FL, and GS: writing—original draft. AH, IM, MAE, MS, RAb, MK, RI, IE, ABa, EA, AAb, MMa, AAh, FA, HM, RAd, SE, MMu, ABr, MIE, MI, and AEA: writing—review and editing. All authors contributed to the article and approved the submitted version.

## Conflict of Interest

GS declares to have received funding support from Biogen, unrelated to this work. The remaining authors declare that the research was conducted in the absence of any commercial or financial relationships that could be construed as a potential conflict of interest.

## Publisher's Note

All claims expressed in this article are solely those of the authors and do not necessarily represent those of their affiliated organizations, or those of the publisher, the editors and the reviewers. Any product that may be evaluated in this article, or claim that may be made by its manufacturer, is not guaranteed or endorsed by the publisher.
