# Supplementary material for: Pathogenic Variants in ABHD16A Cause a Novel Psychomotor Developmental Disorder With Spastic Paraplegia
Source: Front Neurol. 2021 Aug 20;12:720201. doi: 10.3389/fneur.2021.720201 (PMC8417901; doi:10.3389/fneur.2021.720201)
Supplement: Supplementary file 1 [file Data_Sheet_1.docx]

Supplementary figures


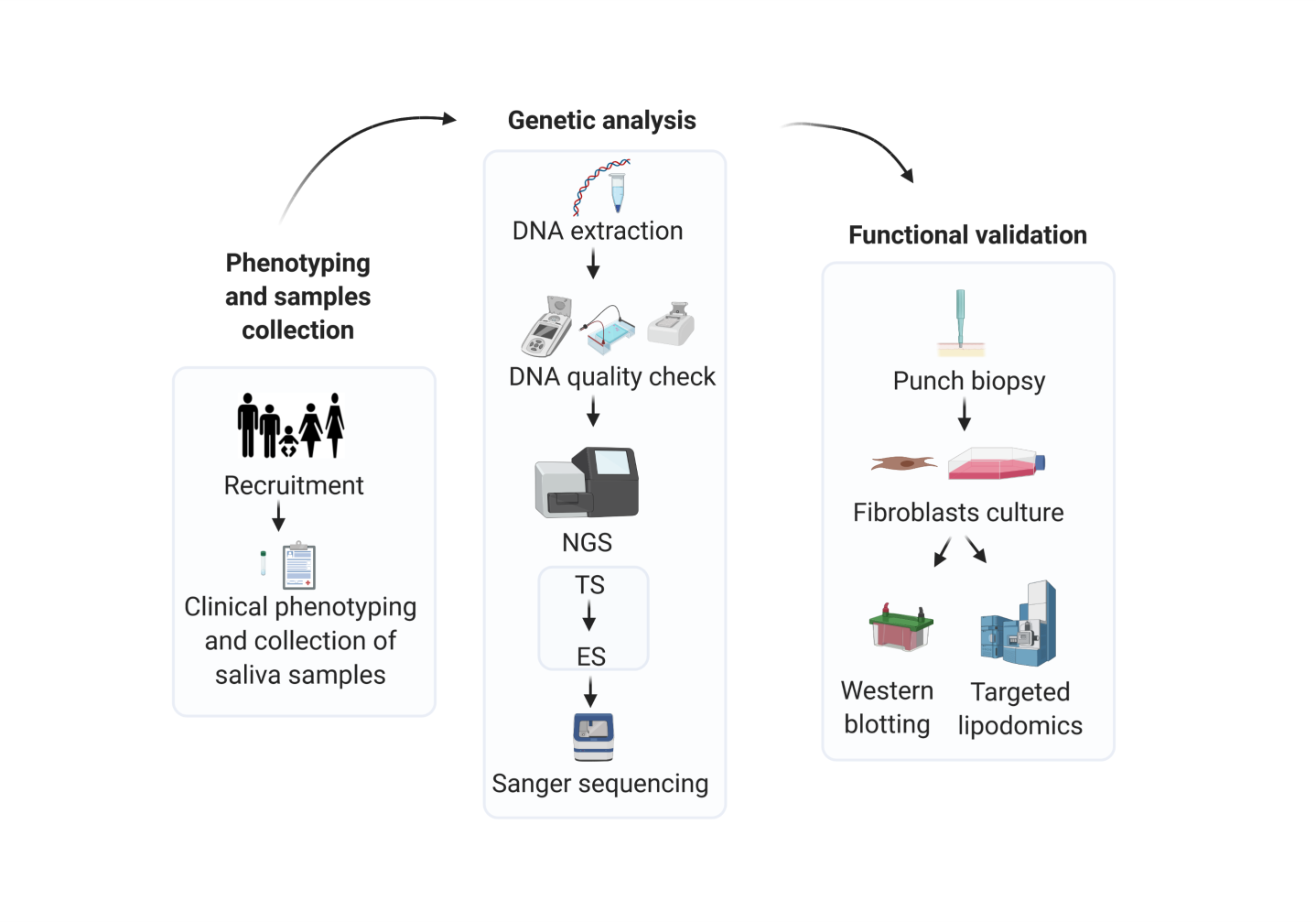


Figure S1. Overview of the tools and approaches we used in this study. NGS, next-generation sequencing; TS, targeted next-generation sequencing panel; ES, whole-exome sequencing.


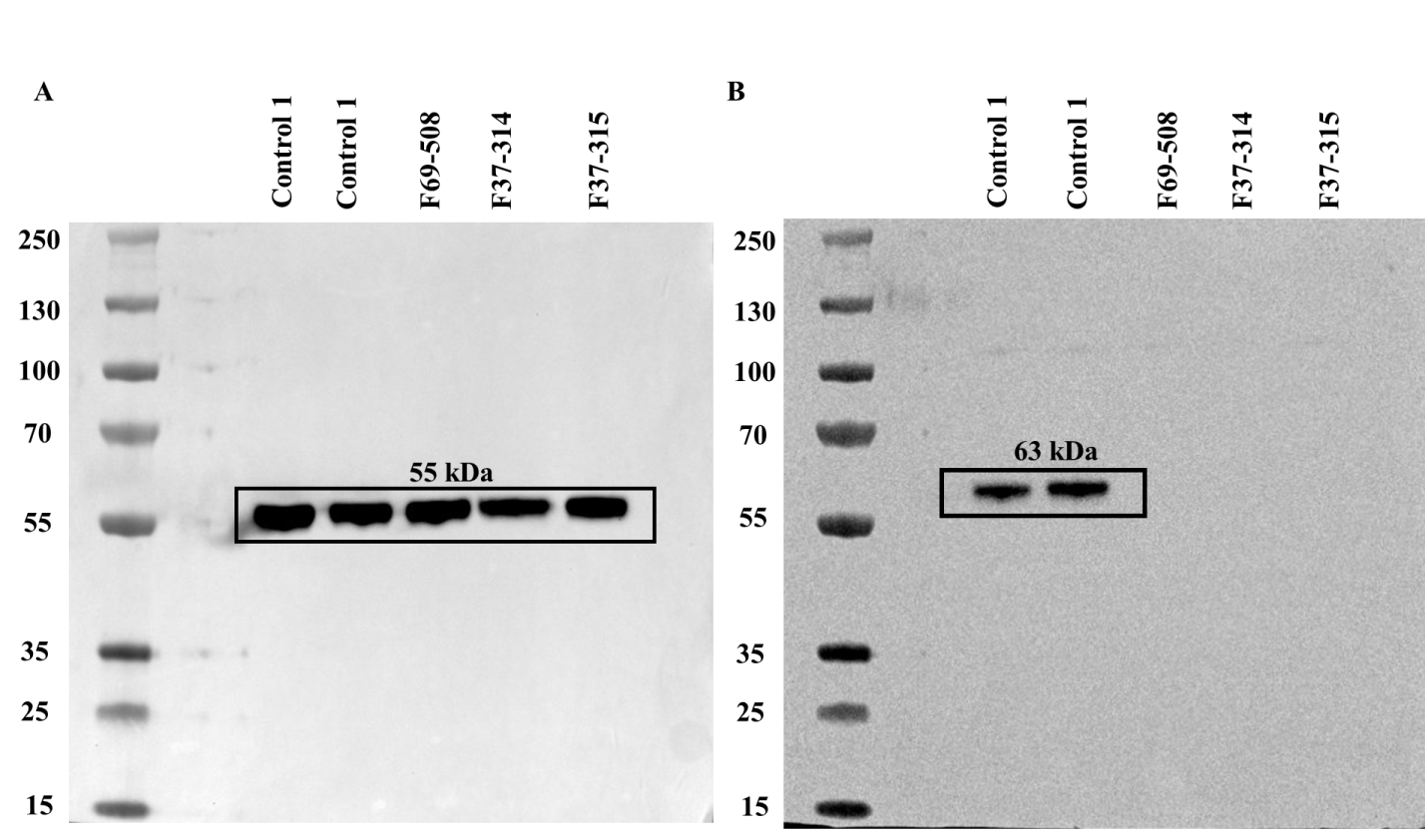


Figure S2. Western blot of proteins extracted from the patients F69-508, F37-314, and F37-315s’ fibroblasts and fibroblasts derived from unrelated healthy controls. Detection of the ABHD16A (B panel) and alpha-tubulin (control protein, A panel) are shown. Sizes in KDa are provided on the left of each panel.

Supplementary tables

| Lipids | Parent (m/z) | Product (m/z) | Cone voltage (V) | Collision energy (eV) | Ionization mode |
| --- | --- | --- | --- | --- | --- |
| Arachidonate | 303,20 | 303,20 | 30 | 0 | Negative |
| LPS 16:0 | 496,2 | 409,2 | 35 | 26 | Negative |
| LPS 17:1 (SI) | 508,37 | 421,50 | 35 | 26 | Negative |
| LPS 18:1 | 522,4 | 435,2 | 35 | 26 | Negative |
| LPS 18:0 | 524,25 | 437,2 | 35 | 26 | Negative |
| LPS 20:0 | 552,2 | 465,2 | 35 | 26 | Negative |
| LPS 22:6 | 568,22 | 481,22 | 35 | 26 | Negative |
| LPS 22:1 | 578,5 | 491,4 | 35 | 26 | Negative |
| LPS 22:0 | 580,2 | 493,2 | 35 | 26 | Negative |
| LPS 24:0 | 608,19 | 521,2 | 40 | 26 | Negative |
| LPC 17:0 | 510,45 | 184,1 | 40 | 25 | Positive |
| PS 16:0/18:1 | 762,46 | 577,3 | 40 | 20 | Positive |
| PS 16:0/18:0 | 764,18 | 579,1 | 40 | 20 | Positive |
| PC 18:0/18 :1 | 788,60 | 605,20 | 40 | 25 | Positive |
| PS 18:0/18:1 | 790,12 | 605,2 | 40 | 20 | Positive |
| PS 18:0/20:4 | 812,7 | 627,7 | 40 | 20 | Positive |
| PS 18:0/20:1 | 814,30 | 629,60 | 40 | 20 | Positive |

Table S1: Parameters used for the detection of specific phosphatidylserine and lysophosphatidylserine species. PS: phosphatidylserine; LPS: lysophosphatidylserine; SI: internal standard.
